# Targeted designed variants of alpha-2-macroglobulin (A2M) attenuate cartilage degeneration in a rat model of osteoarthritis induced by anterior cruciate ligament transection

**DOI:** 10.1186/s13075-017-1363-4

**Published:** 2017-07-25

**Authors:** Yang Zhang, Xiaochun Wei, Shawn Browning, Gaetano Scuderi, Lewis S. Hanna, Lei Wei

**Affiliations:** 1 Department of Orthopedics, the second hospital of the Shanxi Medical University, Taiyuan, China; 2 Cytonics Corporation, 6917 Vista Pkwy N., Suite 14, West Palm Beach, FL 33411 USA; 3 Department of Orthopedics, Alpert Medical School of Brown University/Rhode Island Hospital, Providence, RI USA

**Keywords:** Targeted designed variants of A2M, PTOA, Rat ACLT OA model

## Abstract

**Background:**

The study was performed to evaluate whether targeted alpha-2-macroglobulin (A2M) variants have a similar or enhanced function at wild-type (wt)-A2M to attenuate cartilage degeneration in vivo.

**Methods:**

In and ex-vivo experiment, bovine cartilage explants (BCE) were incubated with TNF-α and IL-1β with or without wt-A2M or A2M variants. Cartilage catabolism was measured in culture supernatant by sulfated glycosaminoglycan (sGAG). In an in-vivo experiment, 2-month-old male Wistar rats (n = 77) were randomly divided into seven groups and treated with different doses of A2M or its variants by intra-articular injection at 24 hours and day 14 after anterior cruciate ligament transection (ACLT), receiving (1) ACLT/PBS; (2) ACLT/wt-A2M (0.153 mg); (3) ACLT/CYT-108 A2M (0.153 mg); (4) ACLT/CYT-108 A2M (0.077 mg); (5) ACLT/CYT-98 A2M (0.153 mg); (6) ACLT/CYT-98 A2M (0.077 mg); or (7) sham/PBS. The joints and synovial lavage were collected 8 weeks after surgery. Fluorescence molecular tomography was used to monitor inflammation in vivo using probes ProSense and MMPSense at 24 hours, and weeks 2, 4, and 6 after surgery. The cartilage damage was quantified using Osteoarthritis Research Society International score and matrix metalloproteinase (MMP)-3, -13, collagen (Col) X, Col 2, Runx2, and aggrecan (Acan) were detected by immunohistochemical analysis (IHC), ELISA, and RT-PCR.

**Results:**

A2M variants inhibited catabolism in the BCE model by up to 200% compared with wt-A2M. ProSense and MMPSense were dramatically increased in all groups after surgery. Supplemental A2M or its variants reduced ProSense and MMPSense compared with the PBS treatment. Less cartilage damage, lower MMP-13 and Col 2 degraded product, and stronger Col 2 synthesis were detected in animals treated with A2M or its variants compared with PBS-treated animals. A2M and its variants enhanced Col 2 and Acan synthesis, and suppressed MMP-3, MMP-13, Runx2, and Col X production. A2M-108 variant demonstrated less cartilage damage compared with wt-A2M and A2M-98 variant.

**Conclusion:**

The targeted variants of A2M have a chondroprotective effect similar to wt-A2M. However, A2M-108 variant has enhanced function to attenuate cartilage degeneration compared with wt-A2M.

## Background

Although osteoarthritis (OA) affects over 40 million Americans, its pathogenesis remains undefined. OA progression is due, at least in part, to the upregulation of inflammation mediators and proteases [1–4]. Since collected evidence has demonstrated that elevated levels of catabolic enzymes in synovial fluid (SF) induce chondrocyte death and cartilage matrix degeneration within one week of joint injury [5–9], early intervention strategies should focus on reducing these cartilage-degrading proteases within a similar time frame. Previous studies have indicated that catabolic proteases and cytokines reach their peak within 48 h after joint injury, which initiates cell death and cartilage matrix degeneration [1]. Thus, early intervention to reduce these catabolic proteases and cytokines is critical to prevent or delay cartilage degeneration.

Evidence from our group [4, 10–12] and others [1, 13] suggests that new molecular interventions targeting these catabolic enzymes can potentially arrest these adverse events and preserve joint health. It is unlikely, however, that blocking only one of these catabolic factors would be enough to repress the multi-catabolic inflammation factors after joint injury.

Our laboratory and others have demonstrated that serum alpha-2-macroglobulin (A2M) is a promising bio-inhibitor for most of these catabolic enzymes, though it is not adequately present in the joint due to the large molecular weight of A2M, which prevents it from migrating into the SF [5, 14–16]. Recently, we have shown that supplemental intra-articular injection of A2M shortly after joint injury provides chondral protection in anterior cruciate ligament (ACL) injury of the knee by reducing these catabolic enzymes [5]. However, administering serum A2M from patients is time-consuming and complex, which may limit clinical application. We designed targeted variants of A2M to enhance A2M inhibitory function to overcome the purification/concentration burden associated with utilizing circulatory wild-type A2M.

Cytonics scientists have synthesized more than 100 variants and tested their inhibition against 12 proteases mostly implicated in cartilage digestion. The two variants that demonstrated the highest inhibitory characteristics toward largest number of proteases were selected; these were variants 98 and 108 (data not shown). In this study, we used bovine articular cartilage explants (BCE) to screen targeted designed A2M variants in vitro, fluorescence molecular tomography (FMT), a new advanced method for measuring certain catabolic protease levels in vivo, histological and immunohistochemical analyses, and RT-PCR, to compare the efficacy of wild-type-A2M (wt-A2M) and two A2M variants in vivo using our established rat anterior cruciate ligament transection (ACLT) OA model. We have clearly shown that cartilage-degrading proteases, such as elastase, Cathepsin G, B, L and S, and matrix metalloproteinase (MMP)-3, MMP-9, and MMP-13, and ADAMTS 4 and 5 are potently inhibited by A2M and its targeted variants in both the BCE model and a surgically induced OA rat model. Supplemental A2M and its variants attenuate cartilage degeneration and inhibit MMP-13 compared with rats treated by PBS. Our evidence suggests that these molecular variants of A2M, especially 108 variant, have a potentially enhanced function relative to wt-A2M to block multiple catabolic proteases and attenuate OA development in the rat ACLT-OA model.

## Methods

### BCE model and treatment

BCE were isolated from heifers 1.0–1.5 years old and were equilibrated for 3 days in culture medium. To degrade cartilage by cytokine treatment, BCE are incubated for 3 days in DMEM containing 10% fetal bovine serum with or without TNF-α 80 ng/ml and IL-1β 8 ng/ml. Cartilage degradation is inhibited with the addition of either a twofold serial dilution standard curve of wt-A2M (2.0–0.13 mg/ml) or A2M variants (0.25 mg/ml) designed to potently inhibit OA. Cartilage catabolism is measured in culture supernatant by sulfated glycosaminoglycan (sGAG) compared to a standard curve of chondroitin sulfate using di-methylmethylamine staining [4, 11, 17].

The bait region of A2M variants was optimized by placing specific sequences around the cleavage sites from the native substrates for ADAMTS 5 and 4, MMPs, elastase and cathepsin. The difference between variables was mainly the order of these sequences. The size of the bait region was the same as the wt and care was taken that the variant sequences maintained a random coil structure similar to the wt sequence (NCBI Reference Sequence: NM_000014.4). Hek293 cells were transiently transfected with plasmid containing the sequence for the new A2M using the Transit Pro Transfection Reagent (Mirus, MIR5700) according to manufacturer’s protocols. Following incubation the conditioned medium was harvested and centrifuged first at 150 rcf for 5 minutes then at 5850 rcf for 20 minutes, to remove cells and debris. High-performance liquid chromatography (HPLC) and ion exchange chromatography were used for initial purification steps. The eluted A2M from the ion exchange column was further purified using cobalt affinity chromatography. The purified A2M was diluted with PBS.

### Rat ACLT OA model and treatment with supplemental intra-articularA2M or injection of its variants

Two-month-old male Wistar rats (n = 77) were purchased from Charles River and randomly divided into seven groups (n = 11/per group) and treated with different doses of A2M and its variants (provided by Cytonics Corp.): (1) ACLT + PBS; (2) ACLT + wt-A2M (0.153 mg); (3) ACLT + CYT-108 A2M (0.153 mg); (4) ACLT + CYT-108 A2M (0.077 mg); (5) ACLT + CYT-98 A2M (0.153 mg); (6) ACLT + CYT-98 A2M (0.077 mg); or (7) sham + PBS. ACLT (groups 1–6) or sham surgery (group 7) were performed on the left knee and the right knee served as an internal control, as published previously [18, 19]. A2M and its variants dissolved in 20 μl of PBS were intra-articularly injected 24 h and 14 days after ACLT. Animals in groups 1 and 7 received an equivalent volume of PBS at identical time points to the experimental groups (2–6) in their left knees to control for any procedural effects. Previous research has demonstrated that there is no statistical difference between ACLT + PBS injection and ACLT without PBS injection in the rat ACLT model. Therefore, we did not compare ACLT + PBS treatment with ACLT without PBS injection in this study. All animals were euthanized at week 8 after the operation.

### Monitoring inflammation dynamically in vivo

FMT is a new, advanced, sensitive, non-ionizing radiation method used to monitor certain catabolic proteases level in real time in vivo [12]. A mix of fluorescent imaging probes ProSense (10 μl, 13.3 μM) and MMPSense (10 μl, 13.3 μM) were injected intra-articularly 24 h after each A2M injection. The fluorescent imaging probes were also injected at weeks 4 and week 6 after surgery on both the right (control) and left knees, respectively. FMT was used to monitor the levels of inflammation in vivo 24 h after injection of ProSense 750 for the detection of plasmin, cathepsin B, L, and S, and MMPSense 680 was used for the detection of MMP-3, MMP-9, and MMP-13. The picomolar concentrations of probes in the knee joint were determined using region of interest analysis. Data are reported as mean ± SE, with 11 animals per group.

### Histologic assessment

The proximal tibiae were removed from the harvested joints and immersed in 10% formalin for 72 h. The specimens were decalcified in 20% ethylenediaminetetraacetic acid solution (pH 7.2). Frontal sectioning was performed. The tibial plateau was cut into two approximately equal pieces, an anterior and a posterior one, along the medial collateral ligament in the frontal plane. The two resulting tissue pieces (anterior and posterior half) were then both embedded in a single paraffin block with the cut planes facing down. The blocks were trimmed to expose the cartilage. Ten adjacent sections were collected at intervals of 0 μm, 200 μm, and 400 μm. Two serial 6-μm-thick sections from each interval were stained with Safranin O. Cartilage degradation was quantified using the Osteoarthritis Research Society International (OARSI) grading system [20]. Three independent observers scored each section blinded, and the scores from the tibial plateau sections were averaged for each individual animal before comparing groups. Our previous study has demonstrated that A2M attenuates cartilage degeneration [5, 17]. In this study, our pre-specified primary outcome measure was to repeat the comparison between the wt-A2M with PBS and then test whether these targeted designed A2M variants can achieve a similar result or even better result than wt using the microscopic OARSI score system [20].

### Immunohistochemical analysis

To detect the distribution of MMP-13, Col 2, Col 2 breakdown product, and Col X in cartilage, 6-μm sections were collected on positively charged glass slides (Thermo Fisher Scientific, Asheville, NC, USA). The sections were dried on a hotplate to increase adherence to the slides. Immunohistochemical (IHC) analysis was carried out using the 3,3′-diaminobenzidine (DAB) streptavidin-peroxidase (SP) DAB Histostain-SP immunohistochemistry kit (ZYMED Laboratories/Invitrogen, Carlsbad, CA, USA). Sections were deparaffinized and rehydrated using conventional methods. Endogenous peroxidase was blocked by treating the sections with 3% hydrogen peroxide in methanol (Sigma-Aldrich) for 30 minutes. The sections were digested by 5 mg/ml hyaluronidase in PBS (Sigma-Aldrich) for 20 minutes. The sections were incubated with specific antibodies against MMP-13 (1:100) (Santa Cruz Biotechnology), types II (1:10) (Developmental Studies Hybridoma Bank, University of Iowa, Iowa City, IA, USA), Col X (1:50) (EMD Biosciences, Billerica, MA, USA) and Col 2 breakdown product (1:100) (IBEX Technologies, Mont-Royal, QC, Canada), respectively, at 4 °C overnight. The negative control sections were incubated with isotype-matched control serum (2 μg/ml) (R&D Systems, Minneapolis, MN, USA) in PBS. Thereafter the sections were treated sequentially with biotinylated secondary antibody and SP conjugate (ZYMED Laboratories/Invitrogen), then developed in DAB chromogen (ZYMED Laboratories/Invitrogen). The sections were counterstained with hematoxylin (ZYMED Laboratories/Invitrogen). Photomicrographs were taken with a Nikon E800 microscope (Nikon, Melville, NY, USA) [17].

#### Rat SF lavage collection and analyses

SF lavages were collected from the knees immediately after euthanasia [4]: 100 μl of isotonic saline solution was injected intra-articularly using a 30-gauge insulin syringe inserted through the inferior patellar tendon [2]. With injection the joint capsule was visibly distended. The knee was then manually cycled through flexion and extension 10 times to distribute the fluid within the joint before collection by joint aspiration. About half of the fluid that was injected was recovered. The SF was centrifuged at 2000 g for 10 minutes to remove cells and debris, and frozen at -80 °C until analysis. MMP-13 was measured in the SF lavage samples by ELISA following the manufacturer’s instructions (Rat MMP13 ELISA Kit Catalog NO.LS-F5518, LifeSpan Biosciences, Inc, WA, USA). Briefly, 100 μl of the standard or the sample were added per well and incubated for 2 h at room temperature (RT). The liquid of each well was aspirated. Then, 100 μl of detection reagent A working solution was added to each well and incubated for 1 h at 37 °C. After washing, 100 μl of detection reagent B working solution was added to each well and incubated for 1 h at 37 °C. Then, 90 μl of substrate solution was added to each well for 30 minutes at 37 °C (protected from light) after washing 5 times. Finally, 50 μl of stop solution was added to each well, and the colorimetric density of the developed plates was determined within 30 minutes using a microplate reader set to 450 nm (Spectramax M2^e^ Multi-Mode Microplate Reader, Molecular Devices, Sunnyvale, CA, USA). The ELISA was performed in duplicate.

#### Real-time PCR (qPCR)

The femoral condyle cartilage was dissected under dissection microscopy. Three rat cartilage samples were pooled (n = 9 per group). Total RNA was isolated using RNeasy isolation kit (Cat. No. 74104, Qiagen, Valencia, CA, USA) [10]; 1 μg of total RNA was transcribed into complementary DNA (cDNA) using the iScript^TM^ cDNA synthesis Kit (Bio-Rad, Hercules, CA, USA). Of the resulting cDNA, 40 ng/ul was used as the template to quantify the relative content of messenger RNA (mRNA) using QuantiTect SYBR Green PCR kit (QIAGEN, Valencia, CA, USA) with the CFX384 Real-Time PCR Detection System (Bio-Rad Laboratories, Hercules, CA, USA). We used rat Col2a1 forward primer - AAG GGA CAC CGA GGT TTC ACT GG, rat Col2a1 reverse primer - GGG CCT GTT TCT CCT GAG CGT; rat Acan forward primer - CAG TGC GAT GCA GGC TGG CT, rat Acan reverse primer - CCT CCG GCA CTC GTT GGC TG; rat Col10a1 forward primer - CCA GGT GTC CCA GGA TTC CC, rat Col10a1 reverse primer - CAA GCG GCA TCC CAG AAA GC; rat Mmp3 forward primer - TTG TCC TTC GAT GCA GTC AG, rat Mmp3 -3 reverse primer - AGA CGG CCA AAA TGA AGA GA; rat Mmp13 forward primer - GGA CCT TCT GGT CTT CTG GC, rat Mmp13 reverse primer - GGA TGC TTA GGG TTG GGG TC; rat Runx2 forward primer - CCGCAC GAC AAC CGC ACC AT, rat Runx2 reverse primer - CGC TCC GGC CCA CAA ATC TC; 18S RNA forward primer - CGG CTA CCA CAT CCA AGG AA, 18S RNA reverse primer - GCT GGA ATT ACC GCG GCT. Relative transcript levels were calculated as

$$ \mathrm{x} = 2{\hbox{-}}^{\Delta \Delta \mathrm{Ct}} $$

in which ΔΔCt = ΔCt E - ΔCt C, and ΔCt E = Ctexp-Ct18S, and ΔCt C = CtC-Ct18S as previously described [5, 17].

#### Statistical analysis

One-way analysis of variance (ANOVA) was used to analyze the differences among mean cartilage damage scoreS, synovial hyperplasia scoreS, FMT scan results, MMP-13 levels, and the mRNA levels of Acan, MMP-3, MMP-13, Runx2, Col2a1, and Col X. The least significant difference (LSD) multiple comparisons test was used to perform pairwise comparisons following the ANOVA. Differences were considered statistically significant at *P* < 0.05. Statistical analyses were performed using SPSS 13.0 software.

## Results

### BCE

**Fig. 1:**
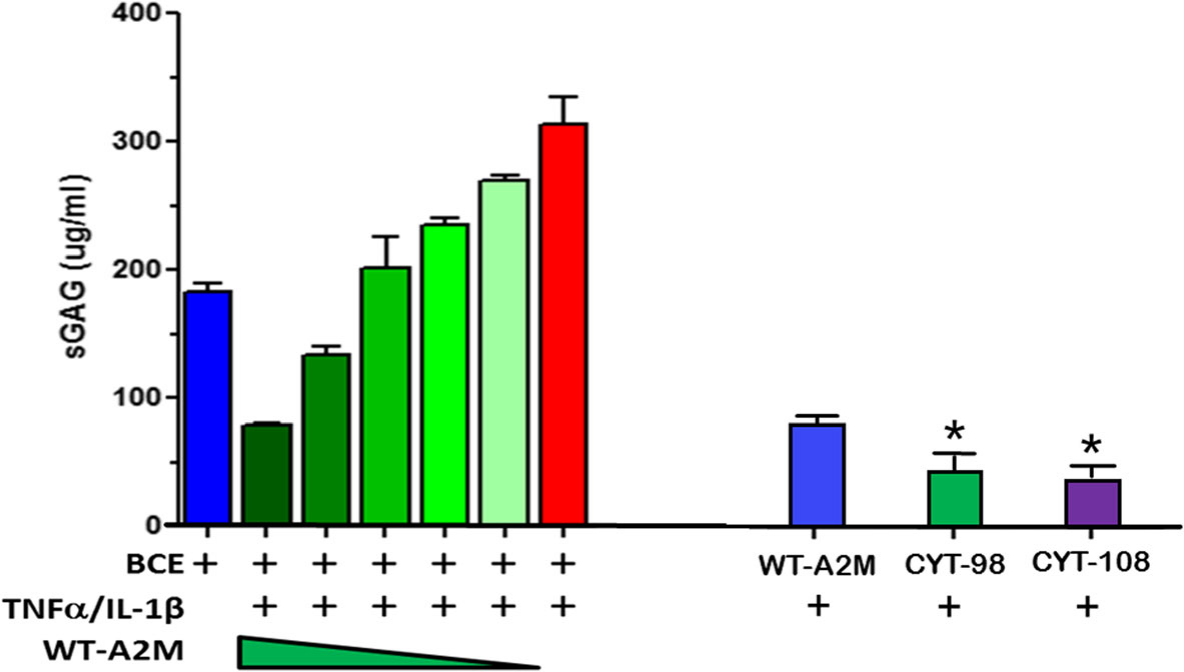
Wild-type alpha-2-macroglobulin (*WT-A2M*) (*left*) and A2M variants CYT-98 and CYT-108 (*right*) inhibit cartilage catabolism induced by TNFα and IL-1β. *Compared with wt-A2M, *P* < 0.05. *BCE*, bovine articular cartilage explants, *SGAG* sulfated glycosaminoglycan

The data obtained from BCE culture supernatant demonstrated that wt-A2M (left) and A2M variants CYT-98 and CYT-108 (right) inhibit cartilage catabolism induced by TNFα and IL-1β. The absolute sGAG values were 313.5 μg/ml in the untreated cytokine-stimulated explants, 78.6 μg/ml in the A2M-treated cytokine-stimulated explants, 44.6 μg/ml in the CYT-98 A2M-treated cytokine-stimulated explants, 37.8 μg/ml in the CYT-108-treated cytokine-stimulated explants, and 183.1 μg/ml in the untreated un-stimulated explants, respectively. The soluble GAG levels determined after treatment with wt-A2M were 1.76-fold and 2.08-fold the levels determined after treatment with variant CYT-98 and CYT-108, respectively. The results also demonstrated that A2M variants were more effective in inhibiting cartilage catabolism in the BCE model by up to 200% compared with wt-A2M (Fig. 1, right) (*P* < 0.05). The variant 108 was the most efficacious inhibitor in the BCE model.

### FMT

**Fig. 2:**
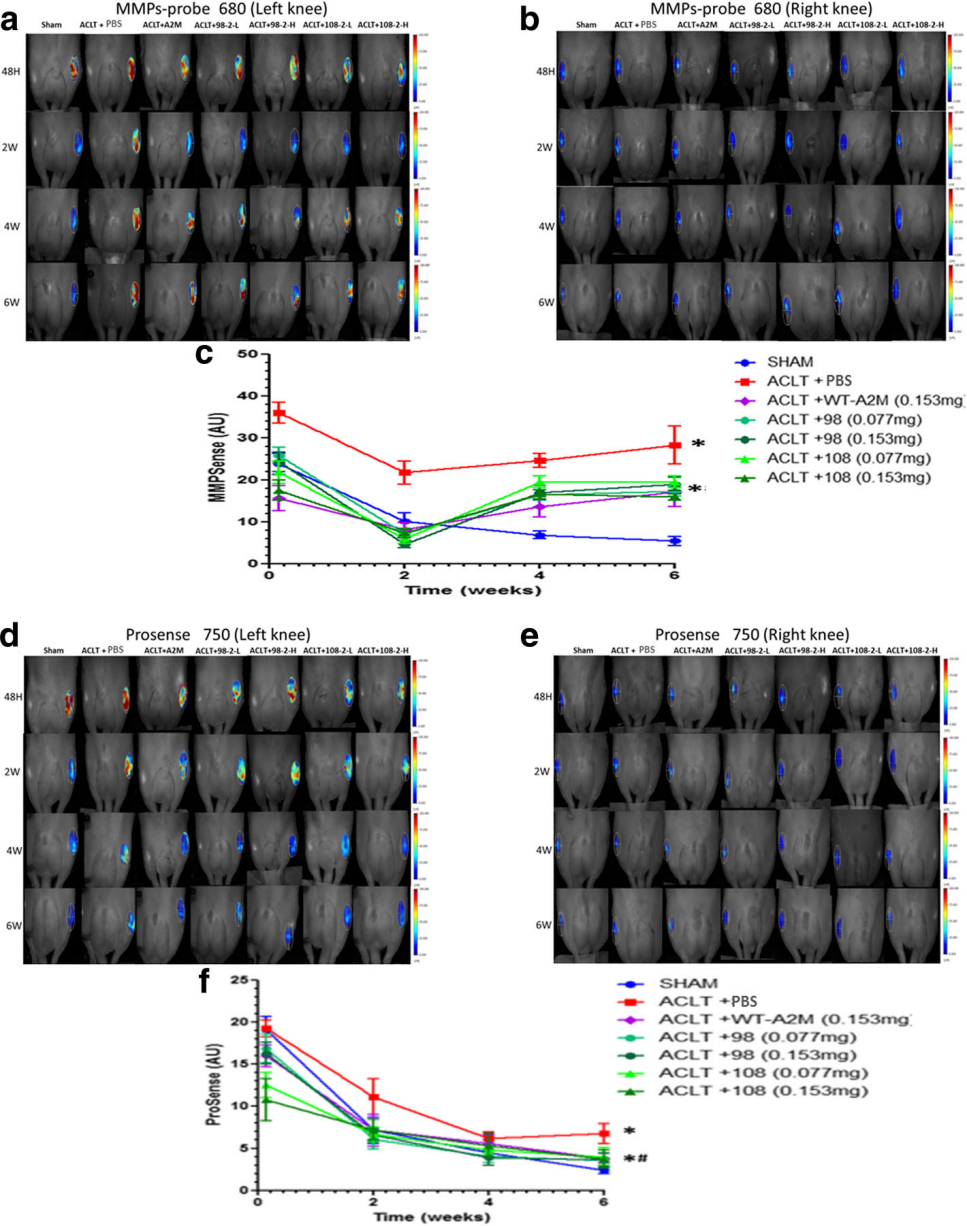
Wild-type alpha-2-macroglobulin (WT-A2M) and its variants CYT-98 and CYT-108 inhibit matrix. metalloproteinases (MMPs) (**a** and **b**) and cathepsin (**d** and **e**). MMPs and cathepsin levels peak 2 days after knee joint injury. The levels of MMPs (**a**) and cathepsin (**d**), as detected by fluorescence molecular tomography after anterior cruciate ligament transection (ACLT), peaked 2 days after surgery, indicating an early catabolic response that subsided thereafter. The mean SD region of interest signal intensities (n = 11 per group) at each time point over a 6-week period are shown (bottom) (**c** and **f**). *Compared with sham, *P* < 0.05. ^**#**^Compared with PBS, *P* < 0.05

Using in vivo deep-tissue imaging methods, real-time information was gained about MMPs and cathepsin biological processes using probes. The levels of ProSense and MMPSense were dramatically increased in all groups after ACLT or sham surgery. MMPs and cathepsin levels peak 2 days after knee joint injury. The highest levels of MMPs (A and B) and cathepsin (C and D), as detected by FMT after ACLT, were observed 2 days after surgery, indicating an early catabolic response. Supplemental A2M or its variants reduced the level of MMPSense (A and B) and ProSense (C and D) compared with the PBS-treated group (Fig. 2, red line) and the sham group (Fig. 2, blue line). This is consistent with our BCE model data. The ProSense remained at a low level at weeks 4 and 6 after surgery even without treatment. Interestingly, at weeks 4 and 6 without treatment, the level of MMPSense was increased again compared with the sham group, but was lower than in the PBS-treated animals. The mean SD region of interest (ROI) signal intensities (n = 11 per group) at each time point over a 6-week period are shown at the bottom (Fig. 2 b and d: *compared with Sham, *P* < 0.05. ^**#**^compared with PBS, *P* < 0.05).

### Supplemental intra-articular injection of wt-A2M and its variants attenuates the severity of OA cartilage degeneration

**Fig. 3:**
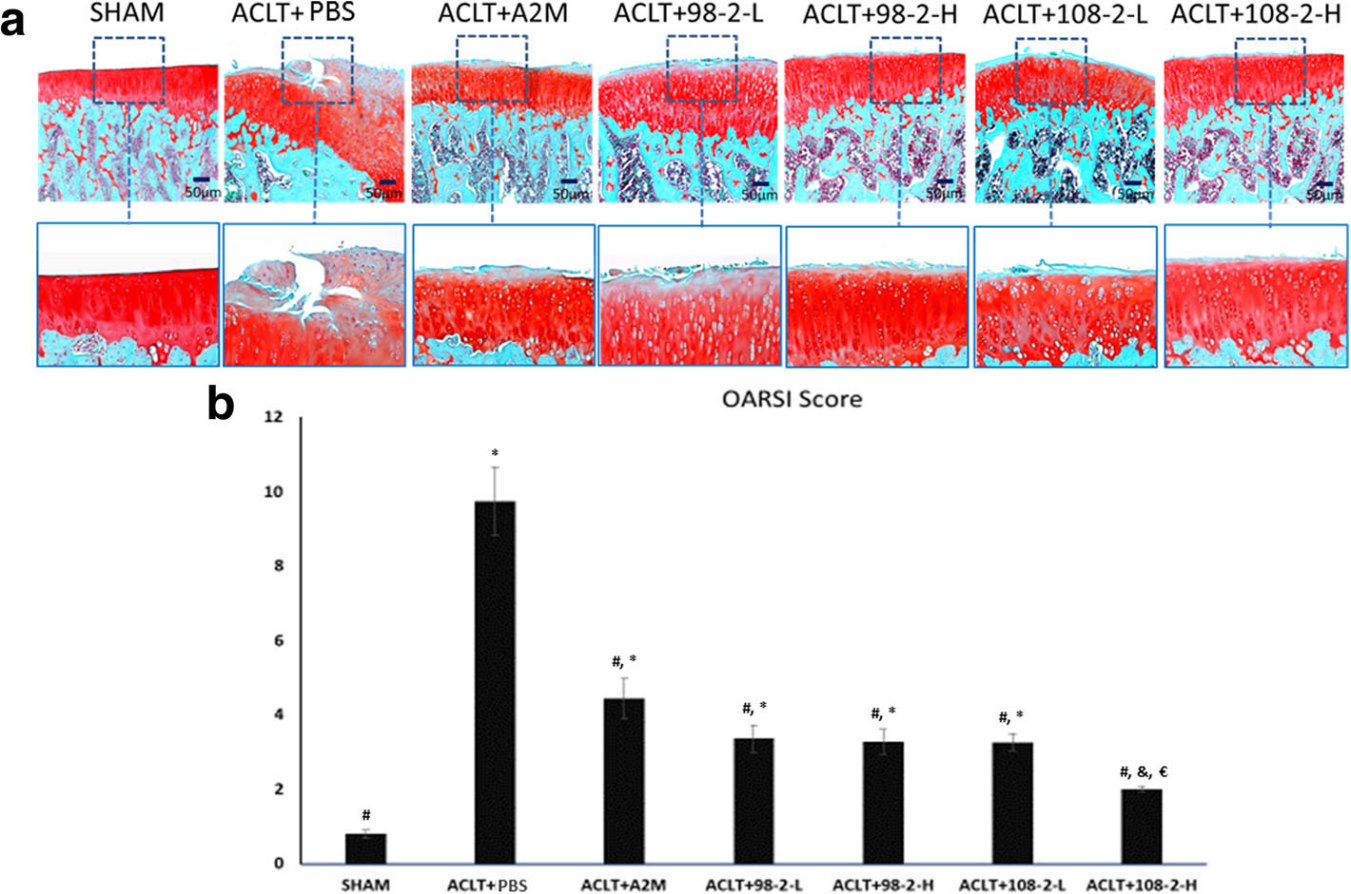
Supplemental intra-articular injection of wild-type alpha-2-macroglobulin (wt-A2M) and its variants CYT-98 and CYT-108 attenuates cartilage degeneration. **a** Strong Safranin O staining and a relatively smoother surface were detected in the articular cartilage from the animals treated with wt-A2M and its variants as compared to PBS-treated controls. **b** The Osteoarthritis Research Society International grading score indicated that cartilage damage was most severe in rats that underwent anterior cruciate ligament transection (*ACLT*) and PBS treatment, while cartilage in rats that underwent sham operation had the least damage. Cartilage damage was further reduced in the rats that received the high dose of the variants of A2M as compared to the rats that received the low dose of the variants of A2M. Values are the mean ± SD. ^**#**^Compared with ACLT + PBS, *P* < 0.05; ^*^compared with sham, *P* < 0.05; ^€^compared with ACLT + A2M, *P* < 0.05

Histological analysis (OARSI score) showed that the animals treated with A2M and its variants had a significant decrease in OA cartilage damage compared with the rats that underwent ACLT and PBS treatment (Fig. 3). Strong Safranin O staining, more cellularity but less chondrocyte cloning, and less fibrillation were observed in the animals treated with A2M and its variants at either concentration compared with the animals treated with the PBS (Fig. 3a). Cartilage in the rats that were treated with A2M variant 108 at high concentration had stronger staining and more intact surface than cartilage in the rats that were treated with variant 108 at low concentration and others, but had weaker staining than the control rats that underwent sham operation. The OARSI grading score indicated that cartilage damage was most severe in rats that underwent ACLT and PBS treatment and the cartilage in rats that underwent sham operation had the least damage (9.75 ± 0.88 and 0.78 ± 0.11, respectively; *P* <0.05), whereas cartilage damage in the A2M and the A2M-variants-treated group was significantly less than in the PBS group (wt-A2M: 4.43 ± 0.52; CYT-98-low: 3.36 ± 0.34; CYT-98-high: 3.27 ± 0.32; CYT-108-low: 3.26 ± 0.21; CYT-108-high: 1.98 ± 0.07; respectively; *P* < 0.01) (Fig. 3b). Values are mean ± SD.

### The animals treated with wt-A2M and its variants had reduced synovial hyperplasia

**Fig. 4:**
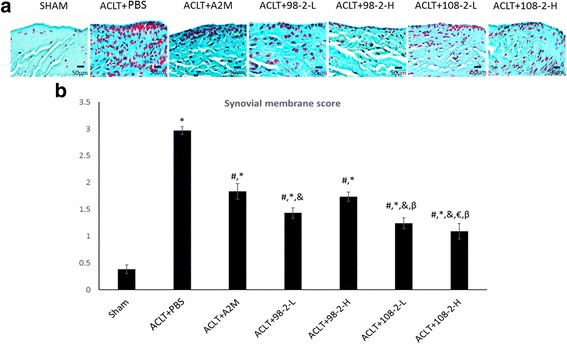
Intra-articular injection of wild-type alpha-2-macroglobulin (wt-A2M) and its variants CYT-98 and CYT-108 leads to a thinner synovial membrane. **a** Hematoxylin/eosin staining indicates the synovium depicted with 1–2 cell layers of synoviocytes in the sham animals. Synovial hyperplasia is seen in the PBS-treated animals with the thicker synovial membranes, whereas the animals treated with A2M and its variants had thinner synovial membranes compared with the PBS-treated animals. Semi-quantified data are shown (**b**). ^**#**^Compared with anterior cruciate ligament transection (*ACLT*) + PBS, *P* < 0.05; *compared with sham, *P* < 0.05; &compared with ACLT + A2M, *P* < 0.05; ^€^compared with ACLT + 98-2-L, *P* <0.05; ^β^compared with ACLT + 98-2-H, *P* < 0.05; ^α^compared with ACLT + 108-2-L, *P* < 0.05

Using hematoxylin/eosin staining, we also observed changes in the synovial membrane in the animals treated with A2M and its variants compared with the animals treated with PBS (Fig. 4a). Only one or two layers of synovial membrane existed in the sham animals. Synovial hyperplasia was seen in the PBS-treated animals with the thicker synovial membranes, whereas the animals treated with A2M and its variants had thinner synovial membranes compared with the PBS treated animals. Semi-quantified data are shown in Fig. 4b.

### IHC analyses

**Fig. 5:**
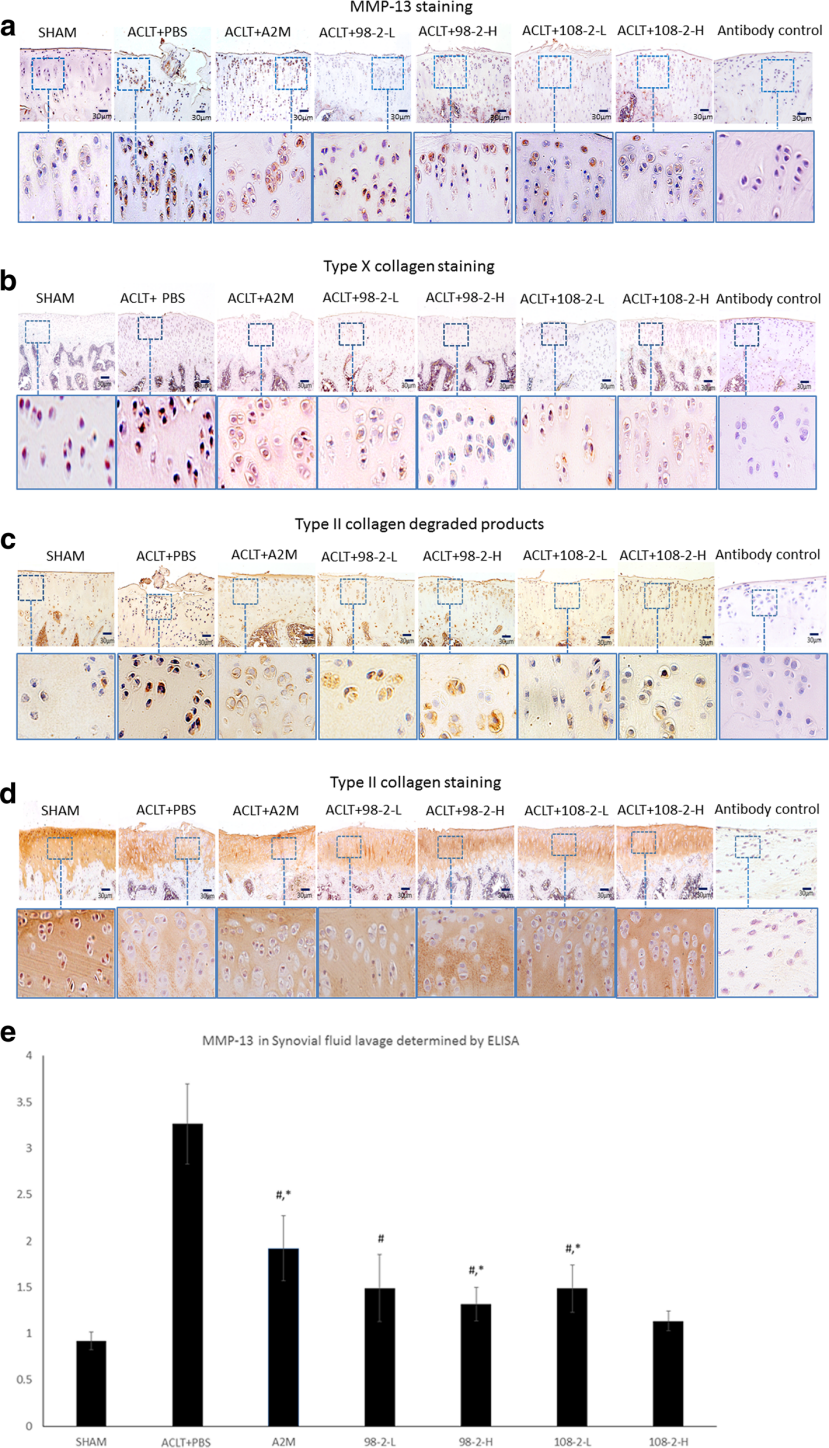
Matrix metalloproteinase 13 (MMP-13) (**a**), type X collagen (**b**), and type II degraded products staining (**c**) were elevated in rats that underwent anterior cruciate ligament transection (*ACLT*) and PBS treatment, but was lower in the alpha-2-macroglobulin (*A2M*) and A2M-variants-treated and sham-operated rats, which is consistent with reduced osteoarthritis damage in these rats. In contrast, type II collagen expression in articular cartilage was higher in the A2M and A2M-variants-treated and sham-operated rats than in rats that underwent ACLT and PBS treatment (**d**). The *bottom panels* are higher-magnification views of the *boxed areas* in the *top panels*. ELISA further confirmed that A2M and its variants partially inhibit MMP-13 (**e**). In A2M and A2M-variants-treated rats, the concentration of MMP-13 in SF was lower than that in the rats that underwent ACLT and PBS treatment but it was still higher than that in in sham-operated rats. Values are the mean ± SD. ^#^Compared with ACLT + PBS, *P* < 0.05; *compared with sham, *P* < 0.05; &compared with ACLT + A2M, *P* < 0.05; ^€^compared with ACLT + 108-2-L, *P* < 0.05

IHC staining showed that matrix metalloproteinase 13 (MMP-13) (Fig. 5a), type X collagen (Fig. 5b), and type II degraded products staining (Fig. 5c) were significantly elevated in rats that underwent ACLT and PBS treatment, but were lower in the A2M-treated and A2M-variants-treated and sham-operated rats, which is consistent with reduced OA damage in these rats (Fig. 3). In contrast, type II collagen expression in articular cartilage was higher in the A2M and A2M-variants-treated and sham-operated rats than in rats that underwent ACLT and PBS treatment (Fig. 5d). The bottom panels of Fig. 3 are higher-magnification views of the boxed areas in the top panels. ELISA further confirmed that A2M and its variants partially reduce the concentration of MMP-13 (Fig. 5e). In A2M and A2M-ariants-treated rats, the concentration of MMP-13 in SF was significantly lower (A2M: 1.92 ± 0.32 ng/ml; CYT-98 low: 1.49 ± 0.33 ng/ml; CYT-98 high: 1.32 ± 0.17 ng/ml; CYT-108 low: 1.49 ± 0.23 ng/ml; CYT-108 high: 1.13 ± 0.10 ng/ml) than that in the rats that underwent ACLT and PBS treatment (3.26 ± 0.40 ng/ml) but it was still higher than that in sham-operated rats (0.92 ± 0.09 ng/ml) (*P* < 0.05). Values are the mean ± SD.

**Fig. 6:**
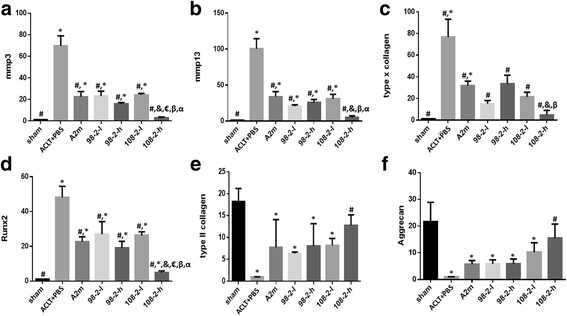
Supplemental intra-articular wild-type alpha-2-macroglobulin (A2M) and its variants CYT-98 and CYT-108 reduce catabolism and enhance anabolic metabolism in the anterior cruciate ligament transection (ACLT) rat osteoarthritis model. The levels of mRNA of the matrix metalloproteinase (MMP)-3(**a**), MMP-13(**b**), type X collagen(**c**), and Runx2(**d**) were expressed at a lower level in rats that were administered A2M and its variants as compared to the rats that underwent ACLT and PBS treatment. In contrast, the levels of mRNA for type II collagen (**e**) and aggrecan (**f**) followed an opposite pattern. Both of them were increased in rats that were administered A2M and its variants as compared to the rats that underwent ACLT and PBS treatment. Values are the mean ± SEM. ^#^Compared with ACLT + PBS, *P* < 0.05 ; *compared with sham group, *P* < 0.05; &compared with ACLT + A2M, *P* < 0.05; ^€^compared with ACLT + 98-2-L, *P* < 0.05; ^β^compared with ACLT + 98-2-H, *P* < 0.05; ^α^compared with ACLT + 108-2-L, *P* < 0.05

Real-time PCR data indicated that **s**upplemental intra-articular injection of wt-A2M and its variants CYT-98 and CYT-108 reduced cartilage matrix catabolism and enhanced anabolic metabolism in the ACLT rat OA model (Fig. 6). The mRNA levels of the MMP-3, MMP-13, Runx2, and Col X were expressed at a lower level in rats that were administered A2M and its variants as compared to rats that underwent ACLT and PBS treatment. In contrast, the levels of mRNA for Col X and aggrecan followed the opposite pattern. Both of them were increased in rats that were administered A2M and its variants as compared to rats that underwent ACLT and PBS treatment, suggesting that A2M has a positive impact on cartilage matrix anabolism. Values are the mean ± SEM. # *P* < 0.05 compared with ACLT + PBS; * *P* < 0.05 compared with sham; &Compared with ACLT + A2M, P < 0.05, € compared with ACLT + 98-2-L, P < 0.05; β Compared with ACLT + 98-2-H, P < 0.05; α Compared with ACLT + 108-2-L, P < 0.05.

## Discussion

As A2M inhibits all classes of endoproteases [5, 21, 22], it could be used to mitigate the progression of OA by neutralizing cartilage catabolic factors. Studies have shown that A2M inhibits the activities of ADAMTS-4, ADAMTS-5, ADAMTS-7, and ADAMTS-12 [21, 22], reduces ligament stump resorption following ACL injury [15], and enhances tendon-bone healing of ACL grafts by inhibiting MMP-13 activity [16]. Thus, the balance of the protease/A2M in vivo may play an important role in mediating cartilage destruction by catabolic enzymes. However, A2M is not present in vivo at sufficient levels to counteract the increased concentrations of catabolic factors that appear after joint injury. The level of A2M in serum is 1.53 mg/ml and the level of A2M in OA SF is 0.24 mg/ml as compared to MMP-13 expression in serum of 91.1 ng/ml and MMP-13 expression in SF of 251 ng/ml [5]. Higher A2M concentration is associated with lower MMP-13 content [5]. The difference is thought to be due to the large molecular weight of A2M, which prevents it’s migration from the blood into the SF [14, 15]. Thus, supplemental A2M may be a potential strategy to attenuate cartilage degeneration by reducing these catabolic enzymes induced by joint injury.

Although autologous A2M is an exciting and potential candidate for OA treatment, clinicians may find the time, training, expense, and potential safety concerns of drawing blood and preparing autologous A2M in an outpatient setting burdensome. An off-the-shelf, optimized recombinant form of A2M and its target variants could eliminate these drawbacks. Our FMT and histology results demonstrated that the recombinant target variants of A2M do attenuate OA damage by inhibiting cartilage-degraded enzymes more effectively than wt-A2M.

FMT is a sensitive bio-imaging method providing non-invasive, deep tissue in vivo imaging and it allows for the evaluation of disease progression at multiple time points without sacrifice of the animal [12, 23–25]. In this study, we found that A2M and its variants are able to specifically inhibit proteases and MMPs either in ex vivo cartilage or in vivo ACLT rat OA models immediately after injury compared to PBS-treated animals. The targeted variants of A2M were more efficacious than wt-A2M in the BCE experiments. A significant decrease of ProSense and MMPSense detected by FMT was observed immediately in the animals treated by A2M and its variants compared with the animals treated with PBS in vivo in the ACLT rat OA model. The level of the ProSense remained at a low level at weeks 4 and 6 after surgery even without treatment. This suggests that ProSense is an acute indicator of inflammation and not sensitive for late stages of OA. This finding is consistent with previous reports [5]. Furthermore, the level of MMPSense increased again gradually when A2M was not re-dosed at weeks 4 and 6. This indicates that MMPs may have two peaks. The first phase appears after the initial trauma to the joint. The second peak is not related to the acute joint injury but is associated with progressive cartilage degeneration. The gradual increase in protease activity suggests to us that a continuing constant supplemental level of A2M may be necessary to prevent catabolic degeneration after joint injury to prevent development of post traumatic OA.

Our histological and biochemistry data further demonstrated that supplemental A2M and its variants not only attenuate cartilage damage and inhibit catabolic factor MMP-13, but also enhance cartilage matrix Col 2 and aggrecan synthesis. The increase in collagen and aggrecan suggests that A2M may have cartilage repair functions or at least does not interfere with cartilage matrix synthesis to proceed. This finding is consistent with a previous report in which a high dose of A2M did not induce chondrocyte death [5]. The findings strongly indicate that A2M and its variants are promising bio-inhibitors for catabolic proteases, and supplemental intra-articular injection of A2M soon after injury may provide a chondral protection effect in vivo in the ACL-injured knee by reducing the presence of local catabolic proteases. Our data also clearly demonstrate these variants of A2M have similar functions compared with wt-A2M. Especially, the targeted 108 variant of A2M has demonstrated a stronger chondroprotective effect to prevent or delay cartilage degeneration compared to wt-A2M and 98 variant.

Collected evidence has demonstrated that A2M has the ability to bind cytokines, such as IL-1b and TNFa and that it also enters cells to regulate cellular responses to other growth factors and cytokines [26–28]. The mechanism by which targeted A2M variants enhance this function is not clear. Since the cytokine binding sites in the A2M variants were not altered, it is very likely these target A2M variants regulate the process through the inhibition of the catabolic enzymes [5, 21, 23–25], but not through binding to the cytokines or altering their function. Further study to explore the strong inhibitory ability of the designed variants to the cytokines compared with wt-A2M is warranted.

One limitation of our study was that the SF samples were obtained from the knees after an injection of 100 μl of saline, and cycling and impartial recovery of diluted fluid. Lavage was required to obtain SF from the rat small joint cavity. All SF measurements should be normalized, i.e. using serum and SF urea. Baseline fluid volume, amongst other factors, might have differed in the SF results. Unfortunately, we did not collect blood samples in this study and were unable to collect enough SF samples, due to the small joint volume, in order to normalize the SF results for the urea experiment. Thus, this MMP-13 data analysis does not preclude other variables such as changes in synovial vascular permeability of protein content with OA onset, although no evidence of joint effusion was noted prior to the lavages.

## Conclusion

Our data show that A2M protects cartilage following joint injuries that can progress to OA. Our engineered, recombinant A2M variants are more protective than the wild-type.

## Abbreviations

A2M: Alpha-2- macroglobulin
ACAN: Aggrecan
ACL: Anterior cruciate ligament
ACLT: Anterior cruciate ligament transection
ANOVA: analysis of variance
BCE: Bovine articular cartilage explants
cDNA: Complementary DNA
Col: Collagen
Ct: Cycle threshold
DMEM: Dulbecco’s modified Eagle’s medium
ELISA: Enzyme-linked immunosorbent assay
FMT: Fluorescence molecular tomography
IHC: Immunohistochemical analysis
IL: Interleukin
MMP: Matrix metallopeptidase
mRNA: Messenger RNA
OA: Osteoarthritis
OARSI: Osteoarthritis Research Society International
PBS: Phosphate-buffered saline
ROI: Region of interest
SF: Synovial fluid
SGAG: Sulfated glycosaminoglycan
TNF: Tumor necrosis factor
wt: Wild-type
